# Complete Sequence of a 641-kb Insertion of Mitochondrial DNA in the *Arabidopsis thaliana* Nuclear Genome

**DOI:** 10.1093/gbe/evac059

**Published:** 2022-04-21

**Authors:** Peter D. Fields, Gus Waneka, Matthew Naish, Michael C. Schatz, Ian R. Henderson, Daniel B. Sloan

**Affiliations:** Department of Biology, Colorado State University, Fort Collins, Colorado, USA; Department of Environmental Sciences, Zoology, University of Basel, Basel, Switzerland; Department of Biology, Colorado State University, Fort Collins, Colorado, USA; Department of Plant Sciences, University of Cambridge, Cambridge, United Kingdom; Department of Computer Science, Johns Hopkins University, Baltimore, Maryland, USA; Department of Plant Sciences, University of Cambridge, Cambridge, United Kingdom; Department of Biology, Colorado State University, Fort Collins, Colorado, USA

**Keywords:** CpG methylation, intracellular gene transfer, numt, nupt, structural variants, tandem duplications

## Abstract

Intracellular transfers of mitochondrial DNA continue to shape nuclear genomes. Chromosome 2 of the model plant *Arabidopsis thaliana* contains one of the largest known nuclear insertions of mitochondrial DNA (numts). Estimated at over 600 kb in size, this numt is larger than the entire *Arabidopsis* mitochondrial genome. The primary *Arabidopsis* nuclear reference genome contains less than half of the numt because of its structural complexity and repetitiveness. Recent data sets generated with improved long-read sequencing technologies (PacBio HiFi) provide an opportunity to finally determine the accurate sequence and structure of this numt. We performed a de novo assembly using sequencing data from recent initiatives to span the *Arabidopsis* centromeres, producing a gap-free sequence of the Chromosome 2 numt, which is 641 kb in length and has 99.933% nucleotide sequence identity with the actual mitochondrial genome. The numt assembly is consistent with the repetitive structure previously predicted from fiber-based fluorescent in situ hybridization. Nanopore sequencing data indicate that the numt has high levels of cytosine methylation, helping to explain its biased spectrum of nucleotide sequence divergence and supporting previous inferences that it is transcriptionally inactive. The original numt insertion appears to have involved multiple mitochondrial DNA copies with alternative structures that subsequently underwent an additional duplication event within the nuclear genome. This work provides insights into numt evolution, addresses one of the last unresolved regions of the *Arabidopsis* reference genome, and represents a resource for distinguishing between highly similar numt and mitochondrial sequences in studies of transcription, epigenetic modifications, and de novo mutations.

**Significance**

Nuclear genomes are riddled with insertions of mitochondrial DNA. The model plant *Arabidopsis* has one of largest of these insertions ever identified, which at over 600 kb in size represents one of the last unresolved regions in the *Arabidopsis* genome more than 20 years after the insertion was first identified. This study reports the complete sequence of this region, providing insights into the origins and subsequent evolution of the mitochondrial DNA insertion and a resource for distinguishing between the actual mitochondrial genome and this nuclear copy in functional studies.

## Introduction

Intracellular DNA transfer from mitochondrial genomes (mitogenomes) into the nucleus is pervasive and ongoing in eukaryotes (Hazkani-Covo et al. 2010). These insertions (known as numts) are usually non-functional and subject to eventual degradation. However, they are of biological interest as a mutagenic mechanism (Turner et al. 2003; Hazkani-Covo and Martin 2017) and the ultimate source of rare functional gene transfers from mitochondria to the nucleus (Timmis et al. 2004). They are also of practical concern as a common cause of artifacts and misinterpretation in inferring phylogenetic relationships (Bensasson et al. 2001), biparental inheritance of mitogenomes (Lutz-Bonengel et al. 2021), and de novo mutations (Wu et al. 2020). Most numts derive from small fragments of the mitogenome, but some can be large and structurally complex, including frequent cases where multiple discontinuous regions of mitochondrial DNA (mtDNA) fuse during integration into the nuclear genome (Portugez et al. 2018).

The initial sequencing of Chromosome 2 in the *Arabidopsis thaliana* genome identified an extremely large numt, which was assembled to be 270 kb in length and represent approximately three-quarters of the 368 kb *Arabidopsis* mitochondrial genome (Lin et al. 1999). However, analysis with fiber-based fluorescent in situ hybridization (fiber-FISH) indicated the assembly of this region was incomplete and estimated an actual size of 618 kb ( ± 42 kb) for the numt (Stupar et al. 2001). This analysis suggested that large regions of repeated sequence were collapsed in the genome assembly, resulting in the erroneous exclusion of the remaining quarter of the mitogenome content that was originally inferred to be absent from the numt. Sequence comparisons between the partial numt and the *Arabidopsis* mitogenome showed high nucleotide sequence identity (99.91%), suggesting an evolutionarily recent insertion, but no evidence of selection to conserve gene function in the numt (Huang et al. 2005).

These early analyses of the Chromosome 2 numt were hampered by multiple technical limitations. It is very difficult with conventional sequencing technologies to accurately assemble regions with long repeats that maintain high sequence identity among copies. More recent efforts to generate complete *Arabidopsis* chromosomal assemblies leveraged advances in long-read sequencing technologies (Naish et al. 2021; Wang et al. 2021), including PacBio High-Fidelity (HiFi), which can produce reads over 15 kb in length with >99% accuracy. These studies were successful in spanning highly repetitive centromere regions, and they both extended the coverage of the Chromosome 2 numt. However, these assemblies differed in multiple regions of the genome (Rabanal et al. 2022), including major disagreements in the length and nucleotide sequence of this numt. The Col-CEN (Naish et al. 2021) and Col-XJTU (Wang et al. 2021) assemblies reported lengths of 370 kb and 641 kb, respectively, and their alignable regions differed by 109 single-nucleotide variants (SNVs), 18 indels, and one 4 bp microinversion even though they were both derived from *Arabidopsis* Col-0 ecotypes.

Another limitation in past analyses of this numt is that the original *Arabidopsis* reference mitogenome (Unseld et al. 1997) and nuclear genome (Arabidopsis Genome Initiative 2000) are derived from different ecotypes (C24 and Col-0, respectively). In addition, the original mitogenome sequence has hundreds of sequencing errors (Davila et al. 2011; Sloan et al. 2018). With the recent generation of accurate long-read sequencing data for the *Arabidopsis* nuclear genome (Naish et al. 2021; Wang et al. 2021) and a reference mitogenome for the Col-0 accession (Sloan et al. 2018), there is a renewed opportunity to assemble and analyze this intriguing numt.

## Results and Discussion

### Structure of the Arabidopsis Chromosome 2 Numt

**Fig. 1.**
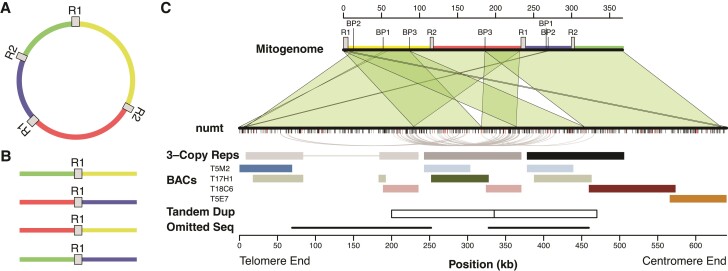
Structure of the *Arabidopsis* chromosome 2 numt. (*A*) A simplified circular representation of the *Arabidopsis* mitogenome. The sequence from the C24 ecotype was used for structural comparisons with the numt because the Col-0 mitogenome contains rearrangements associated with recombination at small repeats (see main text). This conformation of the C24 mitogenome corresponds to the previously described D′–A′–C–B structure (Stupar et al. 2001). R1 and R2 indicate the two large pairs of repeats. Intervening single-copy regions are in different colors, also indicated on the mitogenome map in panel C. (*B*) Recombination between a pair of repeats in the mitogenome produces four possible alternative combinations of flanking sequences (as shown for R1) which are thought to be present at near equal frequencies in tissue samples. The first three of these conformations are all found within the numt. (*C*) Structural comparison of the numt and mitogenome. The mitogenome sequence (top) is annotated with the large repeat sequences (R1 and R2) and pairs of breakpoints (BP1, BP2, and BP3) associated with chimeric fusions in the numt that are possibly the result of non-homologous end joining. Green shaded regions show blocks of syntenic sequence conserved in the numt (bottom). Tick marks below the numt show SNVs (black) and indel/structural variants (red) relative to the Col-0 mitogenome sequence. Some large sections of the mitogenome appear three times in the numt (indicated in shades of gray to black in the 3-Copy Reps row). The curved gray lines connect pairs of variants where two copies share an allele that differs from the mitogenome and the other repeat copy. The colored blocks show locations of four BACs originally used to assemble this genome region. The darker block for each BAC indicates the actual location of that BAC within the numt. The blocks in fainter colors represent repeated sequences similar to the BAC. The adjacent white boxes (Tandem Dup) represent the resulting copies from a putative 135-kb tandem duplication that occurred within the nuclear genome after the numt had already begun to diverge in sequence. The repetitive structure of the numt led to the T17H1 BAC being incorrectly overlapped with the T5M2 and T18C6 BACs in the original *Arabidopsis* genome assembly, resulting in the exclusion of two large regions of intervening sequences (indicated by the black lines in the Omitted Seq row).

By performing a de novo assembly with hifiasm (Cheng et al. 2021) of PacBio HiFi reads generated as part of the recent Col-CEN effort to span the centromeres in the *Arabidopsis* genome (Naish et al. 2021), we produced a gap-free contig that covered the entire numt insertion in Chromosome 2 (fig. 1). The large numt was embedded within a 12.6 Mb contig and was consistent in both size (641 kb) and structure with the recent Col-XJTU genome assembly (Wang et al. 2021), but it differed considerably in nucleotide sequence (see below). Our assembly also matched the repeat structure previously inferred from fiber-FISH and fell within the estimated size range of 618 ± 42 kb from that analysis (Stupar et al. 2001).

The assembled numt is considerably larger than the reference *A. thaliana* Col-0 mitogenome because of extensive sequence duplication, including large tandem repeats. The earlier fiber-FISH study (Stupar et al. 2001) concluded that repeat-mediated overlap between bacterial artificial chromosomes (BACs) used in the original nuclear genome assembly led to the exclusion of a single large internal region. However, by obtaining the entire numt sequence, we found that the T17H1 BAC does not represent the repeat on the centromere-end of the numt as previously inferred. Instead, this BAC derives from the middle of three repeat copies in the numt, meaning that two flanking regions on either side of the T17H1 BAC were omitted from the original assembly (fig. 1*c*).

The numt also exhibits multiple structural differences relative to the mitogenome, including rearrangements arising from recombination between two different pairs of small repeats, which are known as the C and Q repeats and are 457 and 206 bp in length, respectively (Davila et al. 2011) (supplementary fig. S1, Supplementary Material online). Even though the *A. thaliana* nuclear genome sequence derives from the Col-0 ecotype, the conformations associated with these repeat pairs match the *A. thaliana* C24 mitogenome (Unseld et al. 1997). Therefore, the repeat-mediated recombination events that distinguish the Col-0 and C24 mitogenomes likely occurred in the Col-0 mitogenome after the numt insertion, consistent with the relatively rapid accumulation of these rearrangements in the divergence of mitogenome structures among *Arabidopsis* ecotypes (Arrieta-Montiel et al. 2009). However, it is also possible that occasional outcrossing within this largely selfing species (Platt et al. 2010) has led to discordance between the genealogies of the numt and the mitogenome, such that the Col-0 numt is more closely related to the C24 mitogenome than the Col-0 mitogenome.

**Fig. 2.**
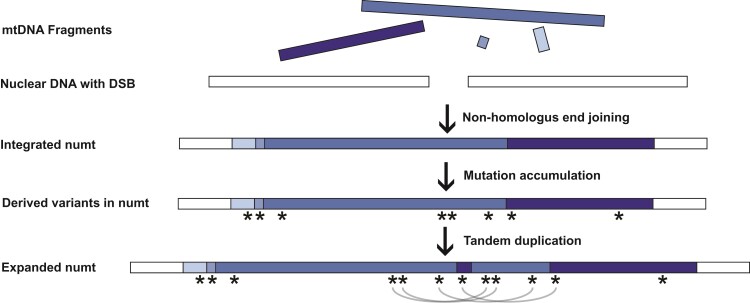
Hypothesized process leading to the origin and evolution of the *Arabidopsis* chromosome 2 numt. The numt appears to have arisen from multiple fragments of mtDNA, including duplicate copies of some regions. These fragments likely integrated into the nuclear genome through non-homologous end joining, followed by the accumulation of sequence variants (indicated by asterisks). A tandem duplication of a large (∼135 kb) region appears to have occurred after numt sequence divergence began based on the presence of shared variants (curved gray lines) between the two duplicate copies of this region. Note that multiple caveats and alternative interpretations apply to this model (see main text). For instance, it is possible that a larger multimeric insertion explains some of the repeated sequence content rather than a fusion of multiple mtDNA fragments at the time of insertion. It is also possible that a complex history of gene conversion between repeated sequences within the numt is responsible for the shared sequence variants.

The *Arabidopsis* mitogenome also contains two pairs of large repeats (6.0 and 4.2 kb in size). In plant mitogenomes, repeats of this size undergo near-constant recombination such that they are present in multiple alternative structures, even within tissue samples (Gualberto and Newton 2017). Three of the four possible alternative conformations associated with the “Repeat 1” pair are found in the numt, meaning that the same flanking sequence can have two different connections on the other side of the repeat (fig. 1). We infer that these alternative structures result from the direct transfer of multiple copies from the mitogenome (fig. 2). Although it is possible that rearrangements generated them within the nucleus after insertion, the fact that the alternative structures already exist at high frequencies within the mitochondria makes direct transfer a much more likely explanation. Therefore, some of the repetitiveness of this complex numt appears to result from the original transfer. Mitogenomes are known to exist in complex structures, including multimeric forms (Bendich 1993), so it is possible that a single transferred molecule could have contained multiple copies of some regions, including these alternative structures. However, complex numts commonly arise via fusion of multiple DNA fragments (Portugez et al. 2018), so it is also possible that the alternative structures were present in distinct DNA fragments that fused at the time of insertion (fig. 2).

Although most of the numt shows conserved synteny with the reference mitogenome or can be explained by repeat-mediated recombination events (see above), there are also structural rearrangements with breakpoints that appear to result from non-homologous end joining (NHEJ). The first 8 kb of sequence at the telomere-end of the numt consists of two fragments from disparate parts of the mitogenome that appear to result from fusion events (BP1 and BP2 in fig. 1*c*). In addition, there is an internal breakpoint in the numt that is not associated with repeat sequences in the mitogenome (BP3 in fig. 1*c*). This novel fusion is duplicated within the numt as part of a large tandem repeat structure. As discussed below, the patterns of sequence divergence among these repeats provide insight into the further expansion of the numt after its original insertion.

### History of Nucleotide Sequence Divergence in the Arabidopsis Chromosome 2 Numt

Even though the structure and length of our numt assembly generally match the corresponding regions in the recent Col-XJTU assembly, the two assemblies differ substantially in sequence. Most notably, the Col-XJTU numt sequence has 260 SNVs relative to our assembly (supplementary table S1, Supplementary Material online). In every one of these cases, the Col-XJTU variant matches the Col-0 mitogenome even in the large regions of the assembly where BACs provide independent validation of our basecalls (fig. 1). Therefore, large portions of the Col-XJTU numt assembly appear to have been “overwritten” by the more-abundant reads derived from the highly similar mitogenome sequence. To further investigate the sequence discrepancies with the Col-XJTU assembly, we performed a de novo assembly of the Col-XJTU HiFi reads, which generated a near-identical sequence (differing by only 5 SNVs) to our de novo assembly of the Col-CEN HiFi reads. Read mapping indicated that these SNVs reflect true differences between the samples used for Col-CEN and Col-XJTU projects (supplementary table S2, Supplementary Material online). Accordingly, the Col-XJTU project identified >1,000 sequence variants and/or errors genome-wide (Wang et al. 2021), suggesting some divergence among the sequenced Col-0 lines.

By comparing the numt to the reference Col-0 mitogenome, we found that they were 99.933% identical in nucleotide sequence (after excluding indels, multinucleotide variants, and short unalignable sequences adjacent to indel regions). This level of sequence identity is even higher than a previously reported value of 99.91% (Huang et al. 2005), which is not surprising because that study was based on only a portion of the numt and a C24 mitogenome reference that was since found to contain numerous sequencing errors. The SNVs that distinguish the numt and the mitogenome are dominated by transitions with GC base pairs in the mitogenome and AT base pairs in the numt (table 1 and supplementary table S3, Supplementary Material online). This signature likely reflects the much higher rate of mutation in the nuclear genome than the mitogenome (Wolfe et al. 1987; Drouin et al. 2008) and the biased mutation spectrum in the nucleus (Ossowski et al. 2010; Weng et al. 2019). SNV transitions showed a bias of 6.7–1 towards AT base pairs in the numt. This bias is approximately twice as strong as previously reported (Huang et al. 2005), indicating that our improved numt assembly and a higher quality mitogenome reference have substantially reduced noise. The sequence divergence between the numt and the mitogenome also showed evidence of a deletion bias in the nuclear genome (Weng et al. 2019), as more than two-thirds of the indels that distinguished the two genomes had the shorter allele in the numt (table 1).

**Table 1:** Sequence Variants Distinguishing the *Arabidopsis* Chromosome 2 Numt from the Col-0 Reference Mitogenome Sequence

Variant	Count
*Total SNVs (Mitogenome<>numt)*	**425**
* Total transitions*	**270**
GC<>AT	235
AT<>GC	35
*Total transversions*	**155**
GC<>TA	58
AT<>CG	30
GC<>CG	42
AT<>TA	25
*Total indels*	**44**
numt shorter	30
numt longer	14

**Fig. 3.**
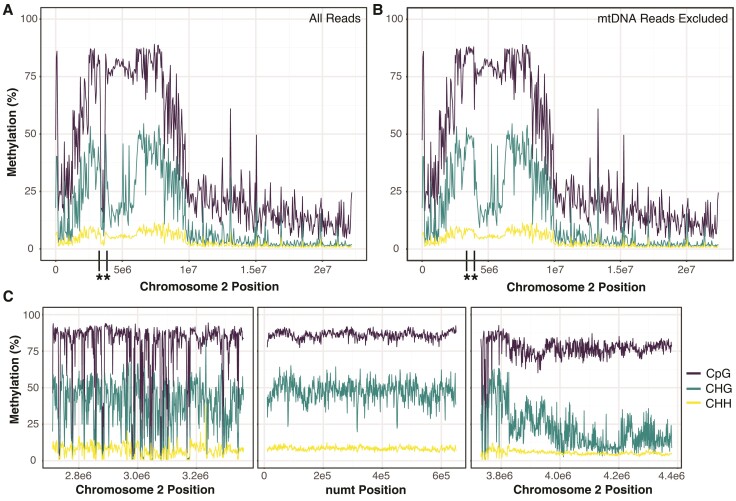
Nanopore-derived estimates of methylation percentage across chromosome 2 of the Col-CEN assembly (after updating it to include the full numt) in CpG (purple), CHG (teal), and CHH (yellow) contexts. (*A*) Methylation profile including all reads (>30 kb) averaged over 50 kb windows. The boundaries of the numt region are indicated with asterisks and vertical black lines on the *x*-axis. (*B*) The same profile after excluding mitogenome-derived reads based on SNVs that distinguish the numt and mitogenome, which greatly increases the estimated methylation levels in the numt because of the lack of methylation in the actual mitogenome. (*C*) Methylation profile of 650 kb on the telomere side of the numt (left) across the numt (middle) and 650 kb on the centromere side of the numt (right) averaged over 1 kb windows.

The C→T transitions that dominate the numt mutation spectrum are a hallmark of the abundant 5-methylcytosine (5mC) modifications at CpG and CHG sites in plant nuclear genomes (Vanyushin and Ashapkin 2011; Weng et al. 2019; Naish et al. 2021; Monroe et al. 2022). We found that 88 of the 235 C→T observed SNVs occur at CpG sites, and an additional 87 occur at CHG sites. This total of 74.5% (175 of 235) represents a highly significant enrichment relative to the 33.3% of all cytosines in the mitogenome that are found in a CpG or CHG context (χ^2^ = 178.9; *P* < 0.0001), supporting the expected role of 5mC modifications in numt sequence divergence. Furthermore, using previously generated nanopore sequencing data (Naish et al. 2021), we found high levels of 5mC modifications across the full-length of the numt, consistent with observations for pericentromeric regions in the rest of the *Arabidopsis* genome (fig. 3). This high level of methylation supports previous conclusions that the numt is likely to be transcriptionally inactive (Huang et al. 2005; Adamo et al. 2008).

The repetitive structure in the numt raises the possibility of a large duplication that occurred during the initial insertion event or one that occurred within the nuclear genome post-insertion. We reasoned that patterns of sequence divergence could differentiate between these alternative models (Hazkani-Covo et al. 2003). If duplicates were generated at the time of insertion, all copies will have started diverging simultaneously and form a “star phylogeny”. In contrast, later duplications within the nucleus after sequence divergence had already begun would lead to descendent copies sharing derived variants with each other. Therefore, we compared sequence divergence among the large repeat regions present in three copies in the numt (fig. 1*c*) and the homologous mitogenome sequence. We found a higher average pairwise divergence between repeats within the numt (0.095%) than between those sequences and the reference mitogenome (0.065%). Again, this is consistent with a higher mutation rate in the nucleus than in the mitogenome. We identified 34 variants for which one of the three copies in the numt matched the mitogenome reference and the other two shared an alternative allele (fig. 1*c*, supplementary table S4, Supplementary Material online). Given the extremely low rate of sequence divergence between repeats, these patterns of shared alleles are highly unlikely to arise by independent mutations (i.e., homoplasy). Instead, they suggest a duplication after nucleotide sequence divergence had already started to occur following the initial numt insertion (fig. 2).

Most of these shared variants occurred in a consistent fashion, supporting tandem duplication of a 135 kb sequence, with a central breakpoint at ∼335 kb from the telomere end of the repeat (fig. 1*c*). However, a cluster of four variants shows a conflicting pattern, linking the internal duplicated region with repeated sequence content at the far telomere end of the numt (fig. 1*c*). These pairings are more difficult to interpret but could reflect a history of localized gene conversion after repeat copies began to diverge. It is also possible that the history of gene conversion is more complex and pervasive across the length of the numt such that shared sequence variants among repeats cannot be used to clearly identify the origins of those copies. Therefore, comparing the divergence of this numt sequence among closely related *A. thaliana* ecotypes may help further tease apart the effects and timing of gene conversion and duplication events. However, other ecotypes sharing this numt insertion have not yet been identified (Pucker et al. 2019; Rabanal et al. 2022).

In summary, the accuracy of PacBio HiFi technology can resolve extremely complex genome structures consisting of long repeats that share highly similar (but non-identical) sequences. *Arabidopsis* is the pre-eminent model system in plant genetics, so obtaining complete and accurate genomic resources is of utmost importance. The original *Arabidopsis* genome assembly (conducted more than two decades ago) (Arabidopsis Genome Initiative 2000) and recent efforts to close the remaining centromere-based gaps (Naish et al. 2021; Wang et al. 2021) represent major landmarks in that process. The resulting PacBio HiFi sequencing data have allowed us to address one of the last remaining unresolved regions in the genome assembly. To our knowledge, this represents the largest numt ever sequenced. Large numt tandem arrays have recently been identified in humans and can reach similar sizes (Lutz-Bonengel et al. 2021), but they have yet to be sequenced. Smaller numt fragments have also undergone massive proliferation into large tandem arrays in legumes (Choi et al. 2022). Insertions of near-complete genomes of plastids and other bacterial endosymbionts have also been observed (Huang et al. 2005; Dunning Hotopp et al. 2007). Therefore, these large insertions are likely common elements of eukaryotic genomes that are frequently overlooked because of challenges associated with assembling regions with such high similarity to organelle/endosymbiont genomes.

Numts are a source of fascination because of their biological importance but also frustration as a source of artifacts in genetic studies. In addition to providing insights into the origins and evolution of this extremely large and complex numt, a complete sequence of this region is of practical value for distinguishing between the numt and true mtDNA in studies investigating molecular processes such as de novo mutation, transcriptional activity, and epigenetic modifications. The similarity of the numt and mitogenome will still pose challenges (especially for short-read sequencing technologies) because stretches of thousands of base pairs remain 100% identical between the numt and the mitogenome, but the set of reliable variants (fig. 1, supplementary table S3, Supplementary Material online) provides a foothold for distinguishing molecular processes associated with these highly similar sequences.

## Materials and Methods

### De Novo Genome Assembly

To generate a de novo assembly of the numt region, we used the full set of PacBio HiFi reads (circular consensus sequences) from Naish et al. (2021), which were accessed via the European Nucleotide Archive (accession number PRJEB46164) on November 18, 2021. We used the hifiasm v. 0.15.1-r334 assembler (Cheng et al. 2021), which was developed for the specific purpose of assembling long, highly accurate reads such as those from PacBio HiFi sequencing. Because the focal genotype is highly inbred, we included the “-l0” flag as part of the assembler configuration, thereby disabling automatic duplication purging. The resultant assembly graph was converted to a set of contigs in a multi-fasta format using AWK as described at https://github.com/chhylp123/hifiasm. To identify the numt region in the resulting contigs we used a local BLAST database and a query composed of the previous, partial assembly of the *A. thaliana* numt sequence. We later repeated these assembly methods with an independent PacBio HiFi data set (Wang et al. 2021), accessed via the Genome Warehouse in the National Genomics Data Center, Beijing Institute of Genomics, Chinese Academy of Science/China National Center for Bioinformation (BioProject PRJCA005809) on November 28, 2021. The structural accuracy of the assembly was validated using multiple orthogonal approaches, including alignment consistency of published Illumina, PacBio HiFi, and nanopore reads mapped to the assembled sequences (Naish et al. 2021; Wang et al. 2021), consistency with the published BAC sequences (Lin et al. 1999), consistency with published fiber-FISH results (Stupar et al. 2001), and consistency with published BioNano optical mapping data (Naish et al. 2021).

### Comparative Sequence Analysis

EMBOSS Stretcher (https://www.ebi.ac.uk/Tools/psa/emboss_stretcher/) was used to generate global pairwise alignments between different assemblies of the numt region. In addition, this aligner was used to compare our assembly to a manually generated rearrangement of the Col-0 mitogenome (GenBank accession NC_037304.1), for which homologous regions of the mitogenome were concatenated to match the synteny of the numt. Multiple sequence alignments of the large repeats in the numt (fig. 1*c*) and homologous mitogenome sequence were generated with MAFFT v7.453 under default parameters (Katoh and Standley 2013). Variants in aligned sequences were identified and quantified with custom Perl scripts. Sequence variants and structural comparisons between the numt, mitogenome, and BACs from the original *Arabidopsis* genome project were visualized with a custom script run in R v4.0.5.

We assessed the quality of basecalls in the de novo numt assembly with local BLAST alignments of the assembly against the numt derived BACs from the original *Arabidopsis* genome assembly and identified seven SNVs distinguishing the de novo assembly and the BACs (supplementary table S5, Supplementary Material online). To validate these seven SNVs, we aligned the HiFi reads to the de novo numt assembly using minimap2 v. 2.22 (Li 2018) and manually inspected the alignments using IGV (Thorvaldsdóttir et al. 2013). For all seven SNVs, the HiFi reads unanimously supported the allele in the de novo numt assembly. We also used the mapped HiFi reads to manually confirm support for five observed SNVs that distinguished our de novo assemblies of the Col-CEN and Col-XJTU HiFi reads (supplementary table S2, Supplementary Material online). The high sequence similarity between the numt and the mitogenome and between large repeats within the numt has the potential to “overwrite” sequence content during assembly, as we observed in the published Col-XJTU assembly (Wang et al. 2021). To confirm that similar errors had not occurred in our hifiasm assemblies, we identified the longest regions in the numt (>7 kb) that shared 100% sequence identity with other numt regions or with the mitogenome. These regions represented the most likely candidates for sequence overwriting. For each region, we extracted spanning HiFi reads that could be anchored to the numt based on flanking variants. The consensus of these reads was then used to confirm the accuracy of the internal sequence.

### Cytosine Methylation Analysis

Previously published nanopore reads (Naish et al. 2021) were filtered for length (>30 kb) using Flitlong (–min_mean_q 95, –min_length 30000; https://github.com/rrwick/Filtlong) and aligned to our de novo Col-CEN numt assembly and the reference Col-0 mitogenome using Winnowmap v1.11, -ax map-ont) (Jain et al. 2020). Alignments were filtered for those containing the numt allele at each SNV position (supplementary table S3, Supplementary Material online) using SplitSNP (https://github.com/astatham/splitSNP). Bam files were merged using Samtools v1.9 and read IDs were extracted and filtered to retain only duplicate IDs (>2). The resulting readset was used for methylation calling against the numt assembly with Deepsignal-plant v0.14 (Ni et al. 2021). Whole-chromosome methylation analysis was performed with the full 30 kb dataset and with the data set generated by removing reads containing mitogenome alleles.

## Supplementary Material

evac059_Supplementary_Data

## Data Availability

All scripts are available via https://github.com/dbsloan/arabidopsis_numt. Alignments and numt sequences are available via https://zenodo.org/record/6168939. The assembled numt sequence (from Col-CEN reads) along with 10 kb of flanking sequence on either side is available on GenBank under accession ON220560.

## References

[evac059-B1] Arabidopsis Genome Initiative . 2000. Analysis of the genome sequence of the flowering plant *Arabidopsis thaliana*. Nature 408:796–815. 11130711 10.1038/35048692

[evac059-B2] Adamo A, Pinney JW, Kunova A, Westhead DR, Meyer P. 2008. Heat stress enhances the accumulation of polyadenylated mitochondrial transcripts in *Arabidopsis thaliana*. PloS One 3:e2889. 18682831 10.1371/journal.pone.0002889 PMC2483354

[evac059-B3] Arrieta-Montiel MP, Shedge V, Davila J, Christensen AC, Mackenzie SA. 2009. Diversity of the Arabidopsis mitochondrial genome occurs via nuclear-controlled recombination activity. Genetics 183:1261–1268. 19822729 10.1534/genetics.109.108514 PMC2787419

[evac059-B4] Bendich AJ . 1993. Reaching for the ring: the study of mitochondrial genome structure. Curr Genet. 24:279–290. 8252636 10.1007/BF00336777

[evac059-B5] Bensasson D, Zhang D-X, Hartl DL, Hewitt GM. 2001. Mitochondrial pseudogenes: evolution's misplaced witnesses. Trends Ecol Evol. 16:314–321. 11369110 10.1016/s0169-5347(01)02151-6

[evac059-B6] Cheng H, Concepcion GT, Feng X, Zhang H, Li H. 2021. Haplotype-resolved de novo assembly using phased assembly graphs with hifiasm. Nat Methods 18:170–175. 33526886 10.1038/s41592-020-01056-5 PMC7961889

[evac059-B7] Choi I-S, et al 2022. Born in the mitochondrion and raised in the nucleus: evolution of a novel tandem repeat family in *Medicago polymorpha* (Fabaceae). Plant J. 110:389–406. 35061308 10.1111/tpj.15676

[evac059-B8] Davila JI, et al 2011. Double-strand break repair processes drive evolution of the mitochondrial genome in Arabidopsis. BMC Biol. 9:64. 21951689 10.1186/1741-7007-9-64 PMC3193812

[evac059-B9] Drouin G, Daoud H, Xia J. 2008. Relative rates of synonymous substitutions in the mitochondrial, chloroplast and nuclear genomes of seed plants. Mol Phylogenet Evol. 49:827–831. 18838124 10.1016/j.ympev.2008.09.009

[evac059-B10] Dunning Hotopp JC, et al 2007. Widespread lateral gene transfer from intracellular bacteria to multicellular eukaryotes. Science (New York, N.Y.) 317:1753–1756. 10.1126/science.1142490 17761848

[evac059-B11] Gualberto JM, Newton KJ. 2017. Plant mitochondrial genomes: dynamics and mechanisms of mutation. Annu Rev Plant Biol 68:225–252. 28226235 10.1146/annurev-arplant-043015-112232

[evac059-B12] Hazkani-Covo E, Martin WF. 2017. Quantifying the number of independent organelle DNA insertions in genome evolution and human health. Genome Biol Evol. 9:1190–1203. 28444372 10.1093/gbe/evx078 PMC5570036

[evac059-B13] Hazkani-Covo E, Sorek R, Graur D. 2003. Evolutionary dynamics of large numts in the human genome: rarity of independent insertions and abundance of post-insertion duplications. J Mol Evol. 56:169–174. 12574863 10.1007/s00239-002-2390-5

[evac059-B14] Hazkani-Covo E, Zeller RM, Martin W. 2010. Molecular poltergeists: mitochondrial DNA copies (numts) in sequenced nuclear genomes. PLoS Genet. 6:e1000834. 20168995 10.1371/journal.pgen.1000834 PMC2820518

[evac059-B15] Huang CY, Grünheit N, Ahmadinejad N, Timmis JN, Martin W. 2005. Mutational decay and age of chloroplast and mitochondrial genomes transferred recently to angiosperm nuclear chromosomes. Plant Physiol. 138:1723–1733. 15951485 10.1104/pp.105.060327 PMC1176441

[evac059-B16] Jain C, et al 2020. Weighted minimizer sampling improves long read mapping. Bioinformatics 36:i111–i118. 32657365 10.1093/bioinformatics/btaa435 PMC7355284

[evac059-B17] Katoh K, Standley DM. 2013. MAFFT multiple sequence alignment software version 7: improvements in performance and usability. Mol Biol Evol. 30:772–780. 23329690 10.1093/molbev/mst010 PMC3603318

[evac059-B18] Li H . 2018. Minimap2: pairwise alignment for nucleotide sequences. Bioinformatics 34:3094–3100. 29750242 10.1093/bioinformatics/bty191 PMC6137996

[evac059-B19] Lin X, et al 1999. Sequence and analysis of chromosome 2 of the plant *Arabidopsis thaliana*. Nature 402:761–768. 10617197 10.1038/45471

[evac059-B20] Lutz-Bonengel S, et al 2021. Evidence for multi-copy mega-NUMT s in the human genome. Nucl Acids Res. 49:1517–1531. 33450006 10.1093/nar/gkaa1271 PMC7897518

[evac059-B21] Monroe JG, et al 2022. Mutation bias reflects natural selection in *Arabidopsis thaliana*. Nature 602:101–105. 35022609 10.1038/s41586-021-04269-6 PMC8810380

[evac059-B22] Naish M, et al 2021. The genetic and epigenetic landscape of the *Arabidopsis* centromeres. Science 374:eabi7489. 34762468 10.1126/science.abi7489 PMC10164409

[evac059-B23] Ni P, et al 2021. Genome-wide detection of cytosine methylations in plant from Nanopore data using deep learning. Nat Commun. 12:5976. 34645826 10.1038/s41467-021-26278-9 PMC8514461

[evac059-B24] Ossowski S, et al 2010. The rate and molecular spectrum of spontaneous mutations in *Arabidopsis thaliana*. Science 327:92–94. 20044577 10.1126/science.1180677 PMC3878865

[evac059-B25] Platt A, et al 2010. The scale of population structure in *Arabidopsis thaliana*. PLoS Genet. 6:e1000843. 20169178 10.1371/journal.pgen.1000843 PMC2820523

[evac059-B26] Portugez S, Martin WF, Hazkani-Covo E. 2018. Mosaic mitochondrial-plastid insertions into the nuclear genome show evidence of both non-homologous end joining and homologous recombination. BMC Evol Biol. 18:162. 30390623 10.1186/s12862-018-1279-x PMC6215612

[evac059-B27] Pucker B, et al 2019. A chromosome-level sequence assembly reveals the structure of the *Arabidopsis thaliana* Nd-1 genome and its gene set. PloS One 14:e0216233. 31112551 10.1371/journal.pone.0216233 PMC6529160

[evac059-B28] Rabanal FA, et al 2022. Pushing the limits of HiFi assemblies reveals centromere diversity between two *Arabidopsis thaliana* genomes. bioRxiv:2022.2002.2015.480579. 10.1093/nar/gkac1115 PMC9757041 36453992

[evac059-B29] Sloan DB, Wu Z, Sharbrough J. 2018. Correction of persistent errors in Arabidopsis reference mitochondrial genomes. Plant Cell 30:525–527. 29519893 10.1105/tpc.18.00024 PMC5894837

[evac059-B30] Stupar RM, et al 2001. Complex mtDNA constitutes an approximate 620-kb insertion on *Arabidopsis thaliana* chromosome 2: implication of potential sequencing errors caused by large-unit repeats. Proc Natl Acad Sci U S A 98:5099–5103. 11309509 10.1073/pnas.091110398 PMC33170

[evac059-B31] Thorvaldsdóttir H, Robinson JT, Mesirov JP. 2013. Integrative Genomics Viewer (IGV): high-performance genomics data visualization and exploration. Brief Bioinform. 14:178–192. 22517427 10.1093/bib/bbs017 PMC3603213

[evac059-B32] Timmis JN, Ayliffe MA, Huang CY, Martin W. 2004. Endosymbiotic gene transfer: organelle genomes forge eukaryotic chromosomes. Nat Rev Genet 5:123–135. 14735123 10.1038/nrg1271

[evac059-B33] Turner C, et al 2003. Human genetic disease caused by de novo mitochondrial-nuclear DNA transfer. Hum Genet. 112:303–309. 12545275 10.1007/s00439-002-0892-2

[evac059-B34] Unseld M, Marienfeld JR, Brandt P, Brennicke A. 1997. The mitochondrial genome of *Arabidopsis thaliana* contains 57 genes in 366, 924 nucleotides. Nat Genet. 15:57–61. 8988169 10.1038/ng0197-57

[evac059-B35] Vanyushin BF, Ashapkin VV. 2011. DNA methylation in higher plants: past, present and future. Biochim Biophys Acta (BBA)-Gene Regulatory Mech. 1809:360–368. 10.1016/j.bbagrm.2011.04.006 21549230

[evac059-B36] Wang B, et al 2021. High-quality *Arabidopsis thaliana* genome assembly with nanopore and HiFi long reads. Genomics Proteomics Bioinf. 10.1016/j.gpb.2021.08.003 PMC9510872 34487862

[evac059-B37] Weng M-L, et al 2019. Fine-grained analysis of spontaneous mutation spectrum and frequency in *Arabidopsis thaliana*. Genetics 211:703–714. 30514707 10.1534/genetics.118.301721 PMC6366913

[evac059-B38] Wolfe KH, Li WH, Sharp PM. 1987. Rates of nucleotide substitution vary greatly among plant mitochondrial, chloroplast, and nuclear DNAs. Proc Natl Acad Sci U S A 84:9054–9058. 3480529 10.1073/pnas.84.24.9054 PMC299690

[evac059-B39] Wu Z, Waneka G, Broz AK, King CR, Sloan DB. 2020. MSH1 is required for maintenance of the low mutation rates in plant mitochondrial and plastid genomes. Proc Natl Acad Sci U S A 117:16448–16455. 32601224 10.1073/pnas.2001998117 PMC7368333

