# Supplementary material for: Complete Sequence of a 641-kb Insertion of Mitochondrial DNA in the Arabidopsis thaliana Nuclear Genome
Source: Genome Biol Evol. 2022 Apr 21;14(5):evac059. doi: 10.1093/gbe/evac059 (PMC9071559; doi:10.1093/gbe/evac059)
Supplement: evac059_Supplementary_Data [file evac059_supplementary_data.zip › SuppMaterial.pdf]

SUPPLEMENTAL MATERIAL

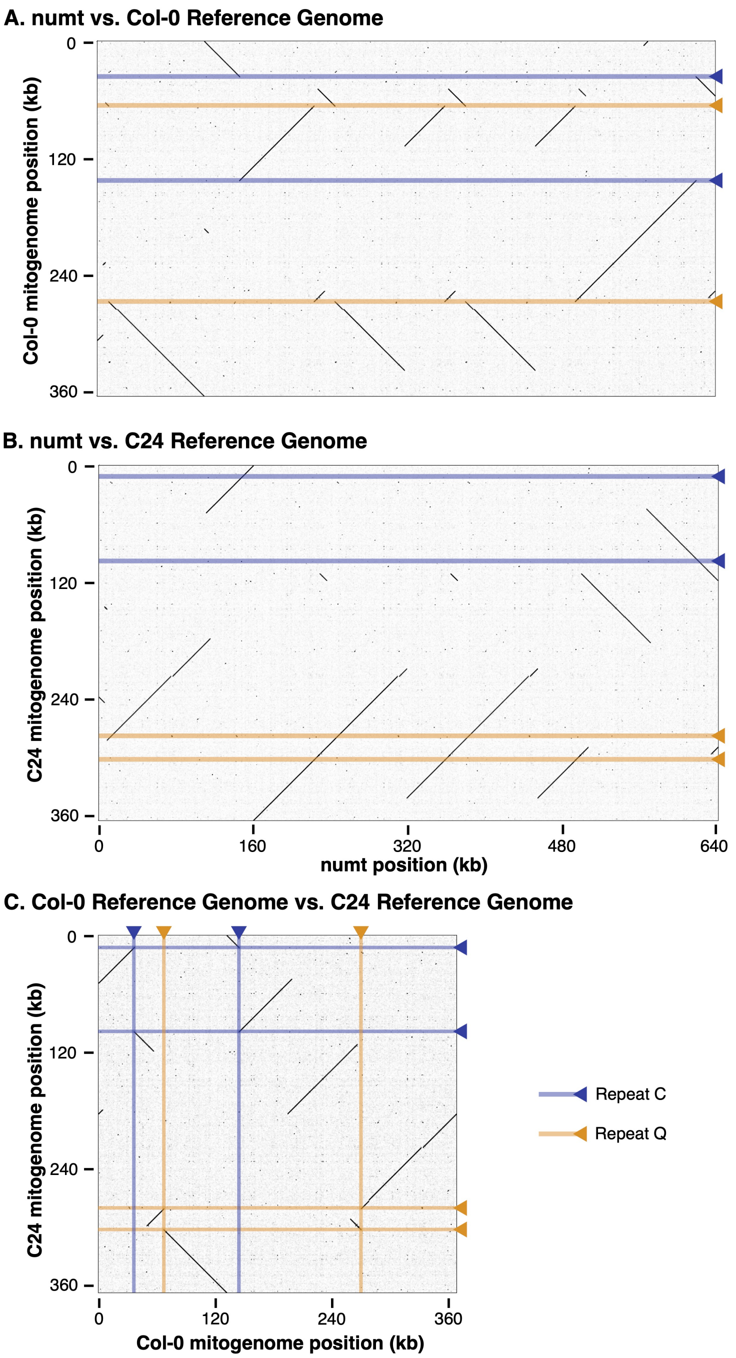

Figure S1. Dot plots comparing structure of the *A. thaliana* Chromosome 2 numt and published reference mitogenomes. (A) Comparison between the numt and *A. thaliana* Col-0 mitogenome (NC_037304.1) and (B) Comparison between the numt and *A. thaliana* C24 mitogenome (Y08501.2). (C) Comparison between the two mitogenomes. Black diagonal lines indicate regions of conserved synteny. The positions of the C and Q repeats in the mitogenome are highlighted in blue and orange, respectively. Note that these two repeats are associated with breaks in conserved synteny with the Col-0 mitogenome due to repeat-mediated recombination but not with the C24 mitogenome. Dot plots were generated with gepard v2.1.0 (Krumsiek, et al. 2007).

Table S1. SNVs that distinguish the numt in our *de novo* assembly of Col-CEN HiFi reads from the corresponding sequence in the published Col-XJTU assembly. Position numbering is relative to the telomere end of the numt in our *de novo* assembly.

Table S2. Variants that distinguish the numt in our *de novo* assembly of Col-CEN HiFi reads from our *de novo* assembly of the Col-XJTU HiFi reads. Position numbering is relative to the telomere end of the numt in the *de novo* Col-CEN assembly.

Table S3. Variants that distinguish the numt in our *de novo* assembly of Col-CEN HiFi reads from the reference Col-0 mitogenome (NC_037304.1). Position numbering is relative to the telomere end of the numt.

Table S4. Pairs of sites in the 3-copy repeats that share a different allele than the mitogenome and the other repeat copy. Position numbering is relative to the telomere end of the numt.

Table S5. Variants that distinguish the numt in our *de novo* assembly of Col-CEN HiFi reads from the sequenced BACs in the original *Arabidopsis* genome project. Position numbering is relative to the telomere end of the numt.
